# Accelerated sarcopenia precedes learning and memory impairments in the P301S mouse model of tauopathies and Alzheimer's disease

**DOI:** 10.1002/jcsm.13482

**Published:** 2024-04-22

**Authors:** Savannah Longo, María Laura Messi, Zhong‐Min Wang, William Meeker, Osvaldo Delbono

**Affiliations:** ^1^ Department of Internal Medicine, Sections on Gerontology and Geriatric Medicine Wake Forest University School of Medicine Winston‐Salem NC USA

**Keywords:** Alzheimer's disease, Denervation, P301S tau mutation, PS19 mouse line, Sarcopenia, Skeletal muscle, Tauopathies

## Abstract

**Background:**

Alzheimer's disease (AD) impairs cognitive functions and peripheral systems, including skeletal muscles. The PS19 mouse, expressing the human tau P301S mutation, shows cognitive and muscular pathologies, reflecting the central and peripheral atrophy seen in AD.

**Methods:**

We analysed skeletal muscle morphology and neuromuscular junction (NMJ) through immunohistochemistry and advanced image quantification. A factorial Analysis of Variance assessed muscle weight, NCAM expression, NMJ, myofibre type distribution, cross‐sectional areas, expression of single or multiple myosin heavy‐chain isoforms, and myofibre grouping in PS19 and wild type (WT) mice over their lifespan (1–12 months).

**Results:**

Significant weight differences in extensor digitorum longus (EDL) and soleus muscles between WT and PS19 mice were noted by 7–8 months. For EDL muscle in females, WT weighed 0.0113 ± 0.0005 compared with PS19's 0.0071 ± 0.0008 (*P* < 0.05), and in males, WT was 0.0137 ± 0.0001 versus PS19's 0.0069 ± 0.0006 (*P* < 0.005). Similarly, soleus muscle showed significant differences; females (WT: 0.0084 ± 0.0004; PS19: 0.0057 ± 0.0005, *P* < 0.005) and males (WT: 0.0088 ± 0.0003; PS19: 0.0047 ± 0.0004, *P* < 0.0001). Analysis of the NMJ in PS19 mice revealed a marked reduction in myofibre innervation at 5 months, with further decline by 10 months. NMJ pre‐terminals in PS19 mice became shorter and simpler by 5 months, showing a steep decline by 10 months. Genotype and age strongly influenced muscle NCAM immunoreactivity, denoting denervation as early as 5–6 months in EDL muscle Type II fibres, with earlier effects in soleus muscle Type I and II fibres at 3–4 months. Muscle denervation and subsequent myofibre atrophy were linked to a reduction in Type IIB fibres in the EDL muscle and Type IIA fibres in the soleus muscle, accompanied by an increase in hybrid fibres. The EDL muscle showed Type IIB fibre atrophy with WT females at 1505 ± 110 μm^2^ versus PS19's 1208 ± 94 μm^2^, and WT males at 1731 ± 185 μm^2^ versus PS19's 1227 ± 116 μm^2^. Similarly, the soleus muscle demonstrated Type IIA fibre atrophy from 5 to 6 months, with WT females at 1194 ± 52 μm^2^ versus PS19's 858 ± 62 μm^2^, and WT males at 1257 ± 43 μm^2^ versus PS19's 1030 ± 55 μm^2^. Atrophy also affected Type IIX, I + IIA, and IIA + IIX fibres in both muscles. The timeline for both myofibre and overall muscle atrophy in PS19 mice was consistent, indicating a simultaneous decline.

**Conclusions:**

Progressive and accelerated neurogenic sarcopenia may precede and potentially predict cognitive deficits observed in AD.

## Introduction

Alzheimer's disease (AD) is the most prevalent form of dementia.^1^ A simultaneous decline in both cognitive function and mobility in aging humans poses a higher risk for dementia compared with either memory or mobility decline alone.^2^, ^3^, ^4^ Hence, factors such as clinical phenotype, peripheral tissue histological profiling, the use of appropriate animal models, biomarkers, and genetics could shed light on specific mechanistic pathways linking accelerated sarcopenia to dementia.

Stronger muscles are associated with a lower risk of AD and mild cognitive impairment.^5^, ^6^, ^7^ Physical function in AD patients declines well before cognitive symptoms emerge,^8^, ^9^ with initial changes in brain regions controlling motor function.^10^, ^11^, ^12^, ^13^, ^14^ Understanding these motor control pathways could lead to better AD treatments.

Interestingly, in AD patients, the loss of lean muscle mass and function precedes brain atrophy and cognitive impairment by several years.^15^, ^16^, ^17^ These patients exhibit decreased physical activity, suggesting that the resulting behavioural alterations could lead to a decline in lean mass. Yet, even when accounting for physical activity levels, lean mass remains independently associated with brain volume. This indicates that the observed decrease in physical activity does not fully explain the accelerated muscle mass and strength loss seen in early AD stages.^18^

Within the conceptual framework of mobility in clinical disease dementia stages, mobility impairments are hypothesized to either predate or follow brain structural alterations.^2^, ^19^ This implies that mobility issues arising at various stages could signify different pathologies.^2^ Our study employed the PS301‐line PS19 mouse, an established model of tauopathies and AD,^20^, ^21^ to ascertain whether motor deficits emerge before, concurrently with, or after a decline in learning and memory throughout the mouse's lifespan.

We hypothesize that diseases commonly classified as dementias, such as AD, are preceded by significant deterioration in skeletal muscle structure, composition, and innervation. The onset of these muscle changes, together with mobility limitations, may serve as early indicators of learning and memory impairments. This could provide a means for earlier detection of central nervous system neurotoxicity, at a stage when the disease remains asymptomatic regarding cognitive functions. This approach will enable implementation of treatment at an early stage of the disease more responsive to proposed treatments.^22^

## Methods

### Mouse model

To understand the involvement of skeletal muscle denervation and motor decline in tauopathies including AD, we used the P301S line PS19 mouse (henceforth PS19). This is a stable C57BL/6 congenic mouse line that expresses the P301S missense mutant form of the human T34 isoform microtubule‐associated protein tau (*MAPT*) 1N4R, under the direction of the mouse prion protein promoter (*Prnp*).^21^ We examined 4 male and 4 female PS19 mice, as well as 4 male and 4 female WT (wild type) mice for each age group (1–2, 3–4, 5–6, 7–8, 9–10, and 11–12 months). The animals were deeply anaesthetised using isoflurane and subsequently decapitated. Postmortem, muscles including the tibialis anterior (TA), gastrocnemius (GA), extensor digitorum longus (EDL), and soleus were dissected. We made every effort to minimize mice suffering. All experimental procedures were conducted in compliance with the National Institutes of Health Laboratory Animal Care Guidelines. The WFUSOM Institutional Animal Care and Use Committee approved Protocol A22–137 for this study.

### Skeletal muscle histochemical and immunofluorescence analyses

For the simultaneous detection of myofibre types and neural cell adhesion molecule (NCAM) expression, we employed a two‐stage histochemical and immunofluorescent staining protocol, consisting of ATPase staining followed by immunostaining for NCAM of the same muscle sections.^23^, ^24^ Myofibre subtype characterization was based on identifying specific myosin heavy chain (MyHC) isoforms following published protocols.^23^, ^24^

### Image analysis

We performed image quantification by using a specialized semiautomatic script designed by our group, which runs within the NIH ImageJ/Fiji software environment.^25^ We measured the cross‐sectional area (CSA) and the number of NCAM positive and negative fibres. Additionally, we assessed fibre grouping, fibre type area, number, and their relative population percentages.

### Neuromuscular junction analysis

For our neuromuscular junction (NMJ) analysis in PS19 and WT mice, we utilized the lumbricalis muscle's unique structure, comprising only a few myofibre layers. This enabled complete imaging of the intact muscle, allowing us to evaluate muscle fibre innervation without the distortions common to cryosectioning.^24^, ^26^, ^27^

### Statistical analysis

To manage the complexity of the data, we applied factorial Analysis of Variance (ANOVA), which allowed us to consider multiple categorical variables simultaneously—genotype, age, and sex—along with a muscle numerical variable. The data underwent rigorous analysis to ensure compliance with the normality and homogeneity of variance assumptions. After identifying significant findings through ANOVA, we conducted post hoc tests to delineate specific group differences. We presented our data as means ± standard error of the mean (SEM). We set up a significance threshold at *P* < 0.05.

Detailed information on methods can be found in the supporting information.

## Results

### Mouse positive genotype exerts significant impact on muscle atrophy with aging

**Figure 1:**
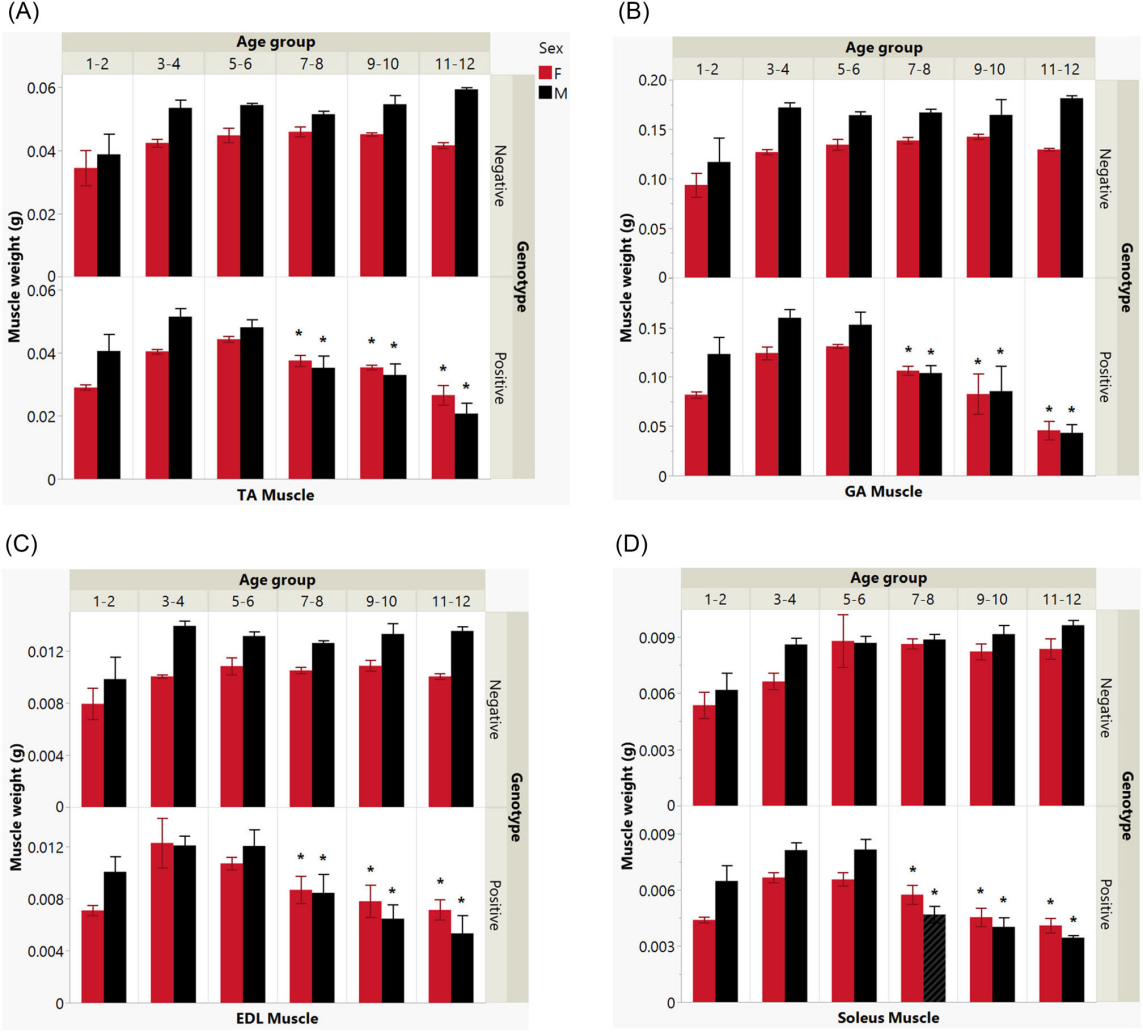
Age and genotype influence on muscle weight in mice differentiated by sex. Bar graphs show muscle weight (in grams) across age groups (1–2 to 11–12 months), categorized by sex (female in red, male in black) and genotype (PS19 positive or WT‐negative). (A) TA muscle, (B) GA muscle, (C) EDL muscle, and (D) soleus muscle. Each bar represents the mean muscle weight, with error bars indicating standard error of the mean. The data reveal a progressive decline in muscle weight due to aging, significantly impacted by the PS19 genotype. No sex differences were observed across any age groups within the studied muscles. Importantly, PS19 positive mice display a unique pattern of muscle weight reduction as they age, distinct from their wild type counterparts. Statistically significant differences are marked by asterisks.

Figure 1 presents a comprehensive view of muscle weights across various age groups while considering the influence of genotype, sex, and muscle type. It highlights the differences in muscle weight resulting from the interplay between age and genotype in both PS19‐positive and WT mice. For the TA muscle, shown in Figure 1A, a statistically significant interaction between age and genotype was noted (*P* < 0.001), indicating that the trajectory of muscle growth with increasing age is influenced by the underlying genotype. At 7–8 months of age, a significant difference in TA muscle weight between WT and PS19 mice was observed for both sexes. In female mice, WT exhibited a weight (in grams) of 0.0459 ± 0.0016 compared with PS19's 0.0375 ± 0.0017 (*P* < 0.001), and in male mice, WT showed a weight of 0.0514 ± 0.0011 versus PS19's 0.0353 ± 0.0037 (*P* < 0.05). This difference became even more marked as the mice aged. The interaction effect between age and positive genotype compared with the negative counterpart was significant (*P* < 0.0001), which suggests that the presence of the PS19 mutation alters the impact of aging on muscle weight. It should be noted that all muscles exhibited increased weight during the first 1–2 months of life due to maturation. Consequently, our analysis employs the data from the 3‐ to 4‐month‐old mice as a baseline for comparison. However, differences in muscle weight by sex within the same age and genotype were not statistically significant, indicating that male and female mice of the same genotype and age have similar muscle weights. This null finding was consistent across all age groups. When examining the interaction between sex and genotype, the data did not reveal a statistically significant relationship (*P* = 0.181). This suggests that the PS19 mutation does not differentially affect muscle weight in male versus female mice. In summary, the results elucidate a significant interplay between age and genotype in determining muscle weight in mice, with the PS19 positive mutation presenting a modifying effect that becomes more pronounced with aging.

In the assessment of the GA, described in Figure 1B, a significant interaction between age and genotype was observed (*P* < 0.00001). Muscle weight is significantly affected by both the age and the presence of the P301S tau mutation in mice. By 7–8 months, muscle atrophy becomes evident in both sexes, with female WT mice having a weight of 0.1385 ± 0.0033 compared with PS19's 0.1065 ± 0.0044 (*P* < 0.001), and male WT mice weighing 0.1669 ± 0.0038 versus PS19's 0.1082 ± 0.0093 (*P* < 0.005). The impact of the genotype on muscle weight was consistent across older age groups, maintaining its statistical significance (*P* < 0.001). The results suggest that the PS19 mutation interacts with age to affect muscle mass in the GA muscle, which becomes more pronounced as the mice age. However, the analysis did not reveal any statistically significant differences in muscle weight between male and female mice within any age group, regardless of genotype. This lack of difference by sex suggests that muscle weight in the GA muscle is not sex‐dependent when controlling for age and genotype. Furthermore, when examining the combined influence of sex and genotype on muscle weight, no statistically significant interaction was detected (*P* = 0.15768). Conclusively, the results highlight a pronounced effect of the interaction between age and genotype on GA muscle weight, with the PS19 positive genotype showing a distinct profile of muscle weight changes with age. However, the effect of sex on muscle weight does not appear to be influenced significantly by the genotype in the GA muscle.

In evaluating the EDL muscle across different age groups, sexes, and genotypes in mice, a statistically significant interaction between age and genotype was demonstrated (*P* < 0.00037) (Figure 1C). The findings demonstrate that muscle weight is influenced not only by the mice's age but also significantly by the presence of the PS19 mutation. Clear signs of muscle atrophy were observed at 7–8 months for both female (WT: 0.0113 ± 0.0005; PS19: 0.0071 ± 0.0008, *P* < 0.05) and male (WT: 0.0137 ± 0.0001; PS19: 0.0069 ± 0.0006, *P* < 0.005) mice. This significant interaction effect persists into older age groups, with *P*‐values consistently below 0.05, indicating the PS19 mutation's pronounced impact on age‐related decrease in EDL muscle weight. In addition to the genotype and age factors, sex also played a significant role in the muscle weight of the EDL muscle. The differences in muscle weight between male and female mice were not statistically significant (*P* > 0.05), indicating that sex does not influence EDL muscle weight. These findings underscore the significant effect of age‐genotype interaction on the EDL muscle weight, with the PS19 positive mutation altering the typical progression of muscle weight changes with age. However, the sex of the mice does not significantly affect EDL muscle weight.

In the analysis of the soleus muscle (Figure 1D), the data revealed a complex interplay between age, genotype, and sex in determining muscle weight. There was a statistically significant interaction between age and genotype (*P* = 0.00718), illustrating that the age‐related changes in muscle weight are influenced by whether the mice are PS19 positive or wild type. At 7–8 months of age, the soleus muscle weight in WT and PS19 mice showed significant differences for both females (WT: 0.0084 ± 0.0004; PS19: 0.0057 ± 0.0005, *P* < 0.005) and males (WT: 0.0088 ± 0.0003; PS19: 0.0047 ± 0.0004, *P* < 0.0001). This difference persisted into the 9‐ to 10‐month and 11‐ to 12‐month age groups, stressing the lasting impact of the PS19 mutation on soleus muscle weight across these ages. This indicates that the PS19 mutation contributes to the differences in muscle weight trajectory as the mice age. In terms of sex differences, the influence on soleus muscle weight was not statistically significant both as a single factor (*P* > 0.05) and when considered in conjunction with age and genotype (*P* > 0.05). These findings suggest that male and female mice exhibit similar patterns of muscle weight across the lifespan, and these patterns are further modulated by the PS19 genotype. Overall, the results for the soleus muscle demonstrate that both the genetic background and mouse age are significant determinants of muscle weight across age groups.

Figure S1 presents a series of scatter plots correlating actual and predicted muscle weights for the TA (A), GA (B), EDL (C), and soleus (D) muscles. The consistency of the *P*‐values across all plots indicates a robust predictive power of the model for each muscle type.

### Denervation, myofibre atrophy, and hybrid myosin heavy chain expression in PS19 mouse muscles

**Figure 2:**
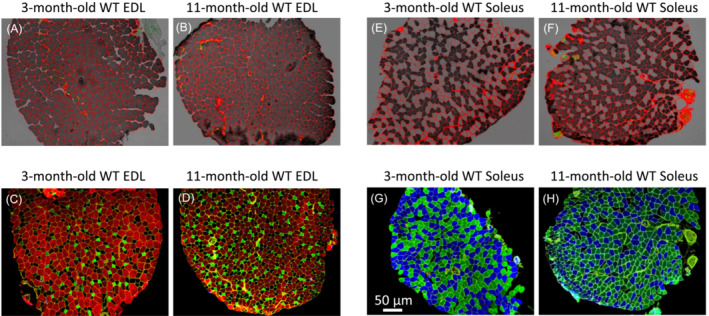
Comparative NCAM immunofluorescence and ATPase staining, plus MyHC and laminin immunofluorescence in EDL and soleus muscles of WT mice. (A) NCAM immunofluorescence and ATPase staining in EDL muscle of 3‐month‐old WT mice. (B) NCAM immunofluorescence and ATPase staining in EDL muscle of 11‐month‐old WT mice. (C) MyHC and laminin immunofluorescence in EDL muscle of 3‐month‐old WT mice. (D) MyHC and laminin immunofluorescence in EDL muscle of 11‐month‐old WT mice. (E) NCAM immunofluorescence and ATPase staining in soleus muscle of 3‐month‐old WT mice. (F) NCAM immunofluorescence and ATPase staining in soleus muscle of 11‐month‐old WT mice. (G) MyHC and laminin immunofluorescence in soleus muscle of 3‐month‐old WT mice. (H) MyHC and laminin immunofluorescence in soleus muscle of 11‐month‐old WT mice.

Figure 2 presents a comparative analysis featuring ATPase histochemical staining, NCAM immunofluorescence, and MyHC with laminin immunofluorescence across various muscle fibre types in the EDL and soleus muscles of 3‐month‐old and 11‐month‐old wild type (WT) mice. The absence of mutated tau expression in the central nervous system results in only modest variations in myofibre cross‐sectional area (CSA), NCAM immunoreactivity, and the MyHC fibre‐type profile.

The examination of EDL and soleus muscle denervation in PS19 mice spanned from 1 to 12 months, representing the initial third of the lifespan in the background C57BL6 mouse strain.^28^ Initially, we highlighted the morphological findings observed at two distinct ages: 3 and 11 months. Subsequently, we delved into the comprehensive analysis spanning the entire lifespan of the PS19 mouse cohort.

**Figure 3:**
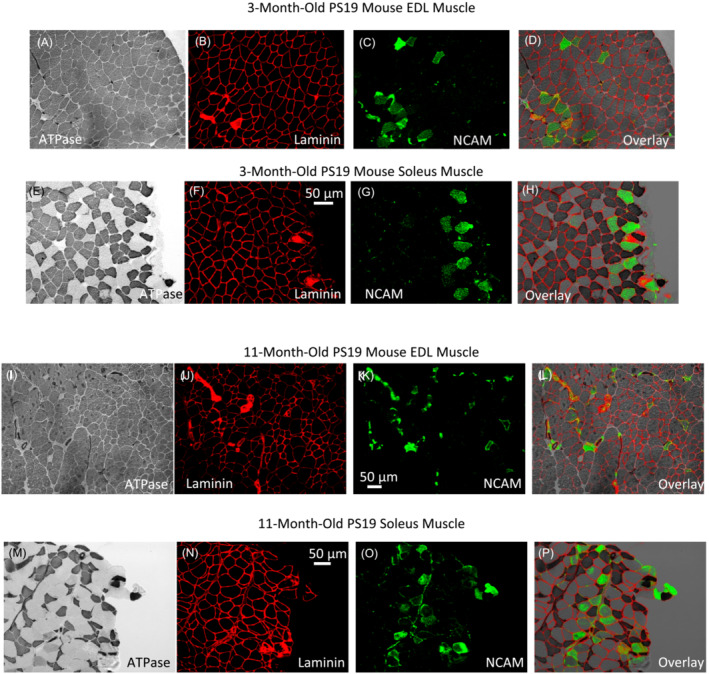
EDL and soleus muscle denervation in 3‐ and 11‐month‐old PS19 mice. (A) EDL muscle cross‐section highlighting its predominant fast‐fibre (Type II MyHC) composition revealed by ATPase staining and observed under brightfield microscopy in a 3‐month‐old PS19 mouse. (B) Laminin staining illustrates the muscle fibre boundaries with a red fluorescence. (C) NCAM expression visualized in green. (D) An overlay combines the A‐C images, showing the localization of fast, Type II myofibres undergoing denervation. (E) Soleus muscle cross‐section showing a mixed fast (Type II, dark)‐ and slow (Type I, light)‐fibre composition revealed by ATPase staining under brightfield microscopy. (F) Laminin staining defined myofibres' boundaries with red fluorescence. (G) NCAM expression in this area, visualized in green, appears more clustered than that in the EDL region. (H) An overlay of images (E)–(G). (I) EDL muscle cross‐section showing its predominant fast‐fibre (Type II MyHC) composition in an 11‐month‐old PS19 mouse. Notice the variability in myofibre cross‐sectional area. (J) Laminin staining showing muscle fibre boundaries in red fluorescence. A thick laminin accumulation is evident in several regions. (K) the expression of NCAM is showed in bright green. Notice the contrasting NCAM distribution: Widespread throughout some fibres while limited to the membrane in others. (L) Overlay of images (I)–(K) integrates the data. It emphasizes the predominant distribution in smaller, atrophic myofibres. (M) Soleus muscle cross‐section reveals a combination of fast (Type II, dark). All images are scaled with a bar representing 50 μm and slow (Type I, light) fibres. The presence of small, angular Type II fibres is clearly discernible by ATPase staining and viewed under brightfield microscopy. (N) Laminin staining for this region reveals the clearly defined myofibre boundaries with red fluorescence and the heterogeneity of myofibres cross‐sectional area. (O) NCAM expression, highlighted in green, exhibits two distinct patterns: A uniform distribution throughout some fibres and a contour pattern in others. (P) An overlay of images (M)–(O) highlights areas where NCAM predominantly overlaps with Type I fibres and mixed fibres (represented in light grey) fibres. For all panels, the reference scale bar represents 50 μm.

The younger group, corresponding to 3‐month‐old PS19 mice shows that the EDL muscle cross‐section consists exclusively of fast‐fibre (Type II MyHC), as was evident by the ATPase staining visualized under brightfield microscopy (Figure 3A). Laminin staining illustrated the defined muscle fibre boundaries, indicative of the homogenous myofibre cross‐section (B). Additionally, neural cell adhesion molecule (NCAM) expression was captured, with its distribution in the tissue clearly highlighted in bright green (C). When images A‐C were integrated, they demonstrated the localization of fast, Type II myofibres undergoing denervation (D). In the soleus muscle, an expected mixed fibre composition was observed. Fast (Type II) fibres appeared dark, while slow (Type I) fibres were lighter (E). Laminin clearly defined muscle fibre boundaries in red fluorescence (F). NCAM expression in the soleus region, represented in green, appeared more concentrated compared with the EDL muscle (G). A composite view of images (E)–(G) emphasized the overlapping regions between NCAM and predominantly Type I fibres (H).

The older group, corresponding to 11‐month‐old PS19 mice shows the EDL muscle cross‐section demonstrating a predominant fast‐fibre (Type II MyHC) composition. Notably, there was a marked heterogeneity in the fibre cross‐sectional area (I). Laminin staining showed the muscle fibre boundaries in red fluorescence, and areas with thick laminin accumulation were distinguishable (J). NCAM expression exhibited a variable pattern, with some fibres showing a widespread distribution, while others presented NCAM limited to the membrane (K). An overlay of images (I)–(K) highlighted the denervation of fast, Type II myofibres, especially emphasizing its prominent distribution in atrophied myofibres (L). The soleus muscle section of the 11‐month‐old mice showed a mix of fast (Type II) and slow (Type I) fibres. Notably, the ATPase staining made evident the presence of small, angulated Type II fibres (M). Laminin staining for this section revealed myofibre boundaries and the variability in the cross‐sectional area of the myofibres (N). The patterns of NCAM expression were dual: some fibres exhibited a homogeneous distribution, while others demonstrated a contour pattern (O). The combination of images M‐O drew attention to the areas where NCAM predominantly overlapped with Type I and mixed (light grey) fibres (P). These results show myofibre denervation and atrophy in both EDL and soleus, muscles that differ in function and myofibre composition.

Next, we sought to elucidate the patterns of NCAM+ myofibre types in EDL and soleus muscles of PS19 mice across different age intervals, genotypes, and sexes.

**Figure 4:**
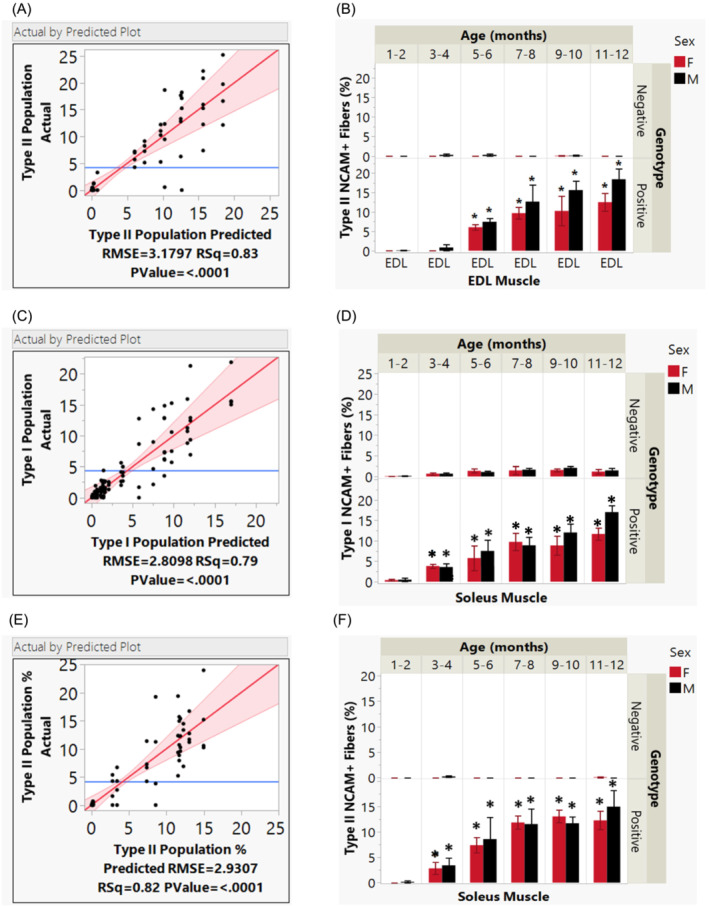
Comparative analysis of NCAM+ myofibre types in the EDL and soleus muscles of PS19 and WT mice over time. Scatter plots (A), (C), and (E) correlate the predicted percentages of NCAM+ fibres with actual observed values, demonstrating the model's predictive reliability. (B) The EDL muscle shows that the proportion of type II NCAM+ fibres is significantly higher in PS19 mice than in WT from as early as 5–6 months (*P* < 0.001), with this difference persisting across their lifespan due to a strong age*genotype interaction (*P* < 0.001). Sex does not show a significant effect (*P* > 0.05). (D–F) The soleus muscle also displays an early and consistent increase in NCAM+ fibres in PS19 mice, starting at 3–4 months, determined predominantly by genotype and its interaction with age, whereas sex alone does not significantly affect the outcome. Factors or interactions with *P*‐values <0.05 are marked with an asterisk (*) to highlight their statistical significance.

Figure 4 presents a complex analysis of NCAM immunoreactive muscle fibre composition in EDL and soleus muscles across genotypes, age, and sex. Graphs A, C, and E illustrate strong correlations in regression analyses between predicted and actual fibre percentages, confirming the model's robustness (*P* < 0.0001). In the EDL muscle (B), the proportion of Type II NCAM+ fibres are significantly higher in PS19 mice than in wild types from as early as 5–6 months (*P* < 0.001), with this difference persisting across their lifespan due to a strong age*genotype interaction (*P* < 0.001). Sex does not show a significant effect on muscle weight, either as an individual factor or in association with genotype and age (*P* = 0.666). The soleus muscle Type I (D) or Type II fibres (F) also exhibits an early and consistent increase in NCAM+ immunoreactivity in PS19 mice, starting at 3–4 months, driven predominantly by genotype and its interaction with age, whereas sex alone does not significantly affect the outcome. Overall, PS19 mice demonstrate an early onset and age‐accelerated increase in NCAM immunoreactivity, predominantly influenced by genotype and age.

### Myofibre distribution, area, and grouping in PS19 mouse muscles

**Figure 5:**
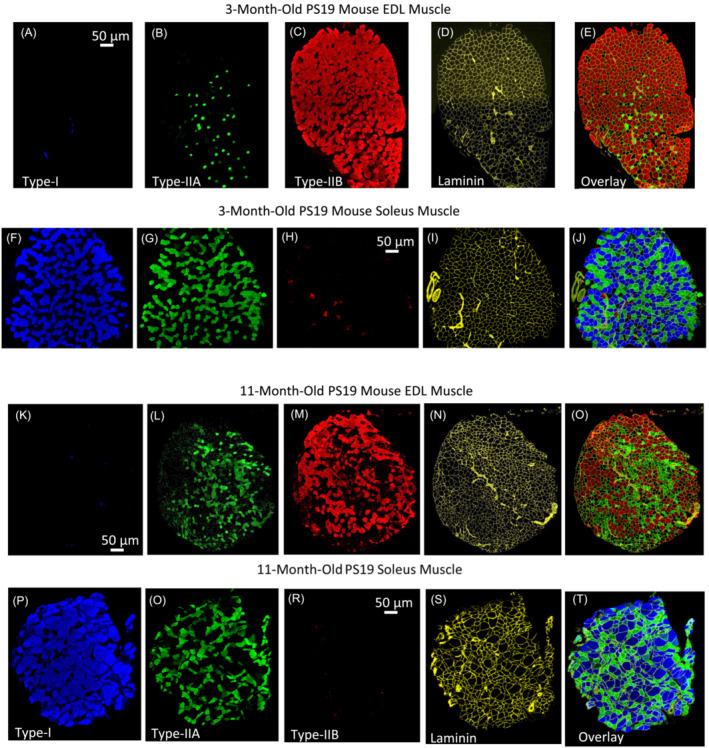
Immunofluorescence staining of different muscle fibre types and laminin in 3‐and 11‐month‐old PS19 mice. Panels (A)–(D) illustrate immunostaining of type I, IIA, IIB, and laminin in the EDL muscle of a 3‐month‐old PS19 mouse. (E) Composite imaging of type I, IIA, IIB fibres and laminin immunostaining. (F)–(J) The images illustrate the immunostaining sequence of type I, IIA, IIB, laminin, and their overlay for the soleus muscle of a mouse of the same age. Note: Type IIX fibres are depicted in black and represent fibres that do not immunoreact to any of the antibodies used. Panels (K)–(N) illustrate immunostaining of type I, IIA, IIB, and laminin in the EDL muscle of a 11‐month‐old PS19 mouse. (O) Composite imaging of type I, IIA, IIB fibres and laminin immunostaining. Panels (P)–(S) illustrate the immunostaining sequence of type I, IIA, IIB, laminin, and their overlay (T) for the soleus muscle of a mouse of the same age. Type IIX fibres are depicted in black and represent fibres that do not immunoreact to any of the antibodies used.

Figure 5 illustrates immunofluorescence staining of different muscle fibre types and laminin in the EDL and soleus muscle of 3‐month‐old PS19 mice. The EDL muscle primarily comprises Type IIA (B) and IIB (C), with an absence of Type I (A) fibres. Overlaying these three fibre types with laminin (D) provides a distinct boundary for the fibres (E), aiding muscle segmentation during digital image quantification. Conversely, the soleus muscle exhibited mainly Type I (F) and Type IIA (G) fibres, with a limited presence of Type IIB (H) fibres. The overlay of MyHC images (F)–(H) plus laminin (I) distinctly displays interspaced Type I and Type IIA fibres (J). Notably, there is no fibre grouping, defined as two fibres of identical phenotype fully surrounded by fibres of the same MyHC type.^29^ Type IIX fibres are depicted represented in black in both muscles, denoting fibres that do not react to any applied antibodies.

Similar to its younger counterpart, there are no type I fibres in EDL muscle from an 11‐month‐old PS19 mouse (K). However, it displays a rise in the number of Type IIA (L) and IIX (O) and a decline in Type IIB (M) fibre. It should be noted that the increase in Type IIA fibres varied among mice, as detailed below. The laminin staining reveals an expanded intercellular space with thicker septa separating the muscle bundles (N). The soleus muscle shows a more disorganized structure with a stark contrast between large type I fibres (P) and the smaller Type II fibres (Q). No Type IIB fibres (R) were detected. The laminin staining (S) highlights myofibre disarray due to the varied fibre cross‐sectional areas, evident in the overlay of image channels (T). It is worth noting the evident atrophy and angulation in Type IIA and Type IIB fibres in the EDL muscle and Type IIA fibres in the soleus muscle. In assessing the accuracy of our predictive model for muscle fibre percentage distribution and cross‐sectional area in PS19 mice, we observed a series of compelling patterns across various fibre types.

**Figure 6:**
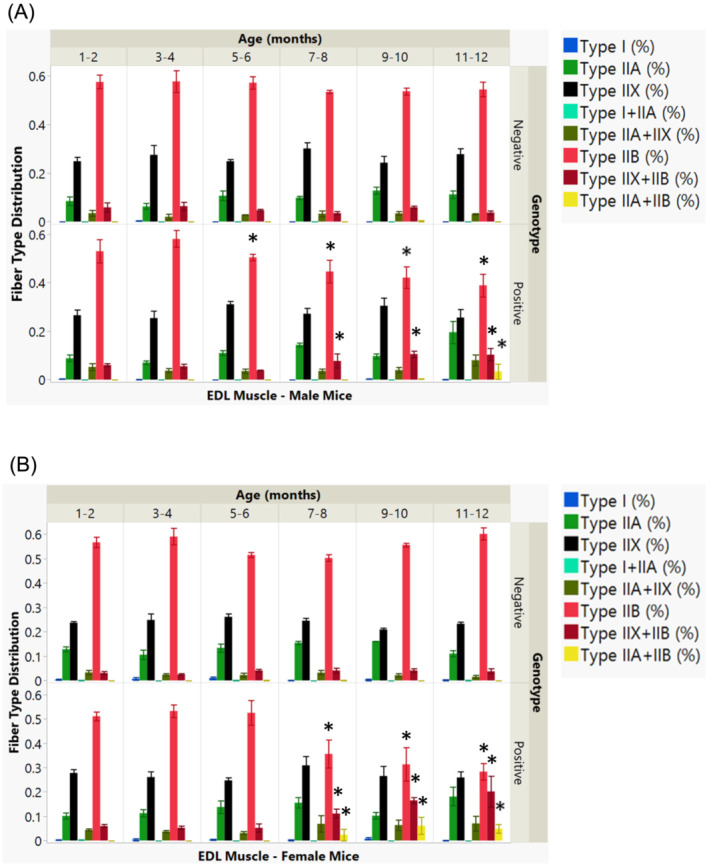
Muscle fibre type distribution in EDL muscle by genotype, age, and sex. (A) The bar graph shows the variation in muscle fibre types within the EDL muscle, categorized by genotype across different age groups in male mice. (*B*) A corresponding graph illustrates the distribution of fibre types in the EDL muscle, also by age and genotype, but focusing on female mice. Distinct colours denote various muscle fibre types, as explained in the accompanying colour key. Statistically significant factors or interactions (*P* < 0.05) are identified with an asterisk (*).

Figure 6A depicts the dynamics of muscle fibre transition in the EDL muscle of male mice, with a notable yet moderate decline in Type IIB fibre population starting at 5–6 months. This reduction is accompanied by a rise in Type IIX + IIB fibres from 7 to 8 months and Type IIA + IIB fibres at 11–12 months. Female mice exhibit a similar trend (B), with a significant decrease in Type IIB fibres emerging later, at 7–8 months, together with increases in Type IIX + IIB and Type IIA + IIB fibres.

**Figure 7:**
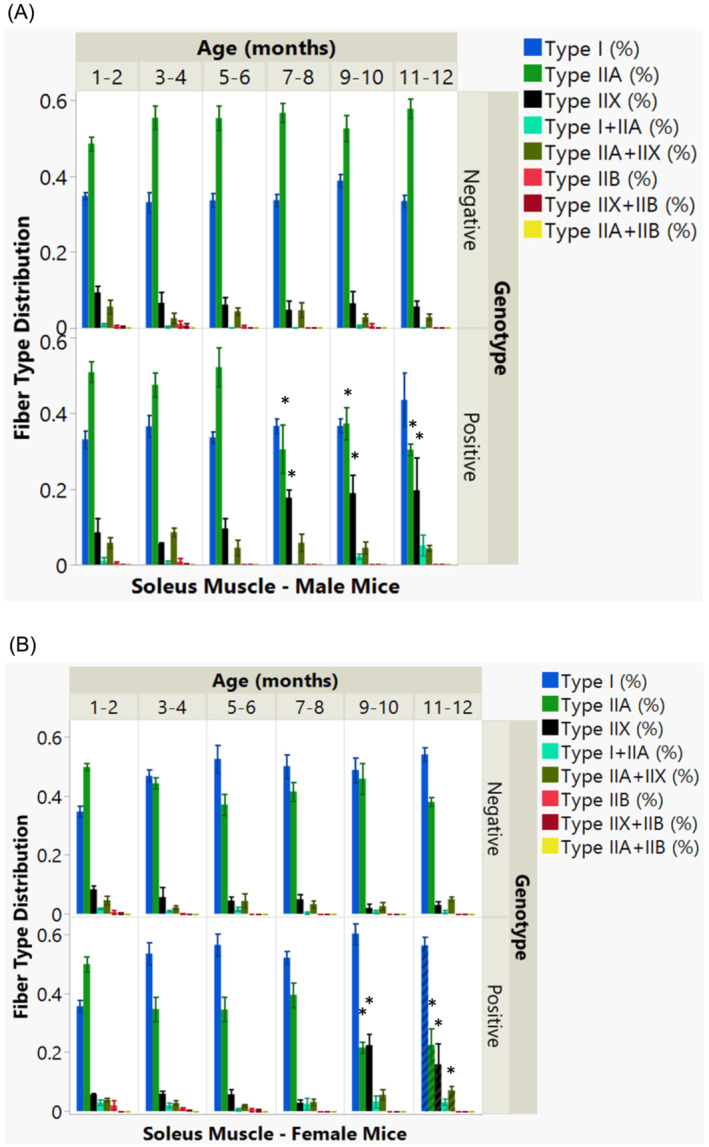
Muscle fibre type distribution in soleus muscles by genotype, age, and sex. (A) The bar graph shows the variation in muscle fibre types within the soleus muscle, categorized by genotype across different age groups in male mice. (*B*) A corresponding graph illustrates the distribution of fibre types in the soleus muscle, also by age and genotype, but focusing on female mice. Distinct colours represent different muscle fibre types, as detailed in the accompanying colour chart. Statistically significant factors or interactions (*P* < 0.05) are identified with an asterisk (*).

In Figure 7, the soleus muscle displays a genotype and age‐related reduction in Type IIA fibres beginning at 7–8 months in male mice, correlating with an increase in Type IIX fibres. Females show an analogous pattern, though onset occurs later, around 9–10 months, with an additional increase in Type IIX and Type IIA + IIX fibres not seen in males.

Overall, the data suggest that genotype and age, along with their interaction, play a pivotal role in the gradual decrease of Type IIB fibres in the EDL and Type IIA fibres in the soleus muscle, with a concurrent rise in hybrid fibres.

In our analysis of the EDL and soleus muscle fibres' CSA, we hypothesized a correlation with the observed age‐dependent muscle mass reduction in PS19 mice. Table 1 illustrates that in WT mice, EDL muscle fibre CSA remains largely unchanged with age. However, PS19 mice exhibit a marked reduction in CSA of Type IIA, IIX, and IIB fibres, with the latter two types showing declines from 5 to 6 months and Type IIA fibres from 7 to 8 months, in both male and female mice. Notably, Type I and hybrid fibres such as Type IIA + IIX or Type IIX + IIB did not demonstrate significant CSA variations.

**Table 1:** Cross‐sectional area of fibre types in EDL muscle

Genotype	Sex	Age (months)	Fibre types CSA (μm^2^)					
			Type I	Type IIA	Type IIX	Type IIA + IIX	Type IIB	Type IIX + IIB
Negative	Male	1–2	0 ± 0	286 ± 25	453 ± 40	305 ± 30	1428 ± 98	792 ± 101
	Female	1–2	165 ± 51	264 ± 16	471 ± 26	202 ± 53	1193 ± 127	771 ± 60
	Male	3–4	34 ± 21	296 ± 6	594 ± 21	218 ± 78	1948 ± 104	1100 ± 64
	Female	3–4	353 ± 281	433 ± 15	739 ± 15	502 ± 166	1508 ± 145	755 ± 219
	Male	5–6	0 ± 0	312 ± 37	623 ± 37	344 ± 45	1690 ± 132	1108 ± 125
	Female	5–6	193 ± 104	296 ± 16	678 ± 43	354 ± 12	1505 ± 110	1137 ± 92
	Male	7–8	0 ± 0	384 ± 32	696 ± 57	416 ± 73	1731 ± 185	1396 ± 201
	Female	7–8	148 ± 148	329 ± 34	709 ± 46	402 ± 58	1578 ± 210	1161 ± 173
	Male	9–10	0 ± 0	333 ± 33	666 ± 35	413 ± 62	1651 ± 105	1082 ± 48
	Female	9–10	85 ± 85	384 ± 28	639 ± 30	523 ± 192	1421 ± 117	846 ± 273
	Male	11–12	0 ± 0	371 ± 26	659 ± 85	341 ± 48	1594 ± 62	1158 ± 166
	Female	11–12	57 ± 57	344 ± 30	557 ± 22	371 ± 48	1415 ± 25	1046 ± 71
Positive	Male	1–2	123 ± 72 (ns)	307 ± 22 (ns)	554 ± 64 (ns)	337 ± 36 (ns)	1488 ± 212 (ns)	924 ± 62 (ns)
	Female	1–2	0 ± 79 (ns)	229 ± 140 (ns)	390 ± 230 (ns)	225 ± 135 (ns)	767 ± 601 (ns)	702 ± 383 (ns)
	Male	3–4	13 ± 13 (ns)	341 ± 22 (ns)	598 ± 50 (ns)	350 ± 24 (ns)	1818 ± 91 (ns)	1059 ± 88 (ns)
	Female	3–4	57 ± 57 (ns)	276 ± 17 (ns)	561 ± 29 (ns)	343 ± 17 (ns)	1208 ± 94 (<0.05)	929 ± 40 (ns)
	Male	5–6	0 ± 0 (ns)	334 ± 28 (ns)	412 ± 44 (<0.01)	330 ± 31 (ns)	1227 ± 116 (<0.05)	1094 ± 106 (ns)
	Female	5–6	128 ± 54 (ns)	305 ± 16 (ns)	400 ± 35 (<0.005)	329 ± 34 (ns)	1037 ± 88 (<0.005)	1104 ± 52 (ns)
	Male	7–8	0 ± 0 (ns)	235 ± 41 (<0.05)	422 ± 44 (0.01)	329 ± 83 (ns)	1121 ± 40 (<0.005)	1099 ± 151 (ns)
	Female	7–8	86 ± 86 (ns)	211 ± 25 (<0.05)	529 ± 54 (<0.05)	386 ± 125 (ns)	1028 ± 42 (0.001)	1103 ± 114 (ns)
	Male	9–10	41 ± 22 (ns)	227 ± 25 (<0.05)	417 ± 42 (<0.001)	360 ± 68 (ns)	987 ± 57 (<0.001)	1327 ± 115 (ns)
	Female	9–10	188 ± 98 (ns)	221 ± 17 (<0.005)	395 ± 60 (<0.05)	326 ± 47 (ns)	1050 ± 31 (<0.05)	925 ± 102 (ns)
	Male	11–12	42 ± 42 (ns)	241 ± 24 (<0.05)	314 ± 75 (<0.05)	280 ± 38 (ns)	822 ± 45 (<0.001)	1472 ± 182 (ns)
	Female	11–12	29 ± 29 (ns)	220 ± 36 (<0.05)	434 ± 27 (<0.05)	318 ± 28 (ns)	1195 ± 132 (<0.05)	799 ± 27 (ns)

Values are expressed as mean ± SEM. Statistical significance is indicated within parentheses. NS indicates no statistically significant differences between genotypes and age groups.

Similarly, to the EDL findings, the soleus muscle in WT mice maintained consistent fibre CSA across the age range (Table 2). Conversely, PS19 mice experienced a pronounced and continuous decrease in the CSA of Type IIA and Type IIX fibres beginning at 5–6 months. This was associated with a decline in the CSA of Type I + IIA and Type IIA + IIX fibres initiating at 7–8 months. Type I fibres remained unaffected. These findings suggest that the expression of mutated tau in PS19 mice precipitates early myofibre atrophy, which exacerbates with advancing age.

**Table 2:** Cross‐sectional area of fibre types in soleus muscle

Genotype	Sex	Age (months)	Fibre types CSA (μm^2^)				
			Type I	Type IIA	Type IIX	Type I + IIA	Type IIA + IIX
Negative	Male	1–2	1067 ± 85	811 ± 96	1078 ± 142	431 ± 159	783 ± 101
	Female	1–2	976 ± 73	650 ± 53	798 ± 129	584 ± 59	611 ± 63
	Male	3–4	1445 ± 108	1433 ± 132	1606 ± 140	425 ± 254	1478 ± 145
	Female	3–4	1350 ± 55	971 ± 33	568 ± 203	695 ± 106	817 ± 117
	Male	5–6	1578 ± 43	1257 ± 43	1511 ± 148	189 ± 189	1149 ± 138
	Female	5–6	1577 ± 44	1194 ± 52	1107 ± 74	729 ± 253	1020 ± 187
	Male	7–8	1543 ± 220	1315 ± 47	1367 ± 62	495 ± 51	1522 ± 162
	Female	7–8	1574 ± 183	1003 ± 57	1157 ± 94	681 ± 81	865 ± 82
	Male	9–10	1481 ± 105	1330 ± 92	1610 ± 145	713 ± 39	1143 ± 58
	Female	9–10	1539 ± 73	1136 ± 49	735 ± 42	606 ± 62	920 ± 36
	Male	11–12	1390 ± 69	1253 ± 27	1452 ± 119	712 ± 43	1148 ± 46
	Female	11–12	1121 ± 71	987 ± 74	852 ± 41	787 ± 22	911 ± 39
Positive	Male	1–2	1022 ± 84 (ns)	743 ± 117 (ns)	777 ± 71 (ns)	244 ± 141 (ns)	819 ± 159 (ns)
	Female	1–2	1065 ± 76	648 ± 41	665 ± 37	602 ± 106	651 ± 65
	Male	3–4	1460 ± 70 (ns)	1172 ± 80 (ns)	1290 ± 73 (ns)	410 ± 149 (ns)	1071 ± 109 (ns)
	Female	3–4	1317 ± 103	910 ± 77	888 ± 92	927 ± 54	887 ± 120
	Male	5–6	1478 ± 33 (ns)	1030 ± 55 (<0.05)	1141 ± 26 (<0.05)	121 ± 121 (ns)	1239 ± 49 (ns)
	Female	5–6	1445 ± 57 (ns)	858 ± 62 (0.01)	762 ± 40 (<0.05)	459 ± 78 (ns)	777 ± 60 (ns)
	Male	7–8	1471 ± 72 (ns)	1013 ± 55 (<0.01)	1119 ± 43 (<0.05)	337 ± 32 (<0.05)	950 ± 61 (<0.01)
	Female	7–8	1314 ± 129 (ns)	752 ± 62 (<0.05)	636 ± 91 (<0.05)	428 ± 41 (<0.05)	547 ± 68 (<0.05)
	Male	9–10	1557 ± 108 (ns)	941 ± 41 (<0.05)	973 ± 139 (<0.05)	497 ± 32 (<0.001)	704 ± 71 (<0.005)
	Female	9–10	1462 ± 58 (ns)	658 ± 73 (<0.01)	304 ± 31 (<0.001)	359 ± 27 (<0.05)	560 ± 61 (<0.005)
	Male	11–12	1331 ± 147 (ns)	691 ± 81 (<0.005)	687 ± 57 (<0.005)	412 ± 72 (<0.05)	792 ± 51 (<0.005)
	Female	11–12	1210 ± 211 (ns)	650 ± 102 (<0.05)	514 ± 125 (<0.05)	458 ± 43 (<0.005)	751 ± 44 (<0.05)

Values are expressed as mean ± SEM. Statistical significance is indicated within parentheses. NS indicates no statistically significant differences between genotypes and age groups.

Our analysis of myofibre grouping in the EDL muscle of PS19 and wild type mice showed no significant differences, regardless of sex. Table S1 indicates that Type I fibre grouping was absent in both male WT and mutant mice. Although Type I and Type II fibre grouping occurred in female mice, comparisons between PS19 and WT mice across age groups failed to show any statistically significant differences.

### Neuromuscular junction denervation with genotype and aging

To understand the relationship between the reduction of pure MyHC type myofibres and the increase in hybrid myofibres, along with a notable and progressive decrease in muscle fibre cross‐sectional area (CSA) and an increase in NCAM immunoreactivity, we hypothesized that these changes might be attributed to denervation at the neuromuscular junction (NMJ). To investigate this, we utilized lumbricalis muscles from 2‐, 5‐, and 10‐month‐old PS19 mice due to their fewer myofibre layers compared with other muscles like the EDL or soleus, which enhances NMJ staining and optical penetration for confocal microscopy.

**Figure 8:**
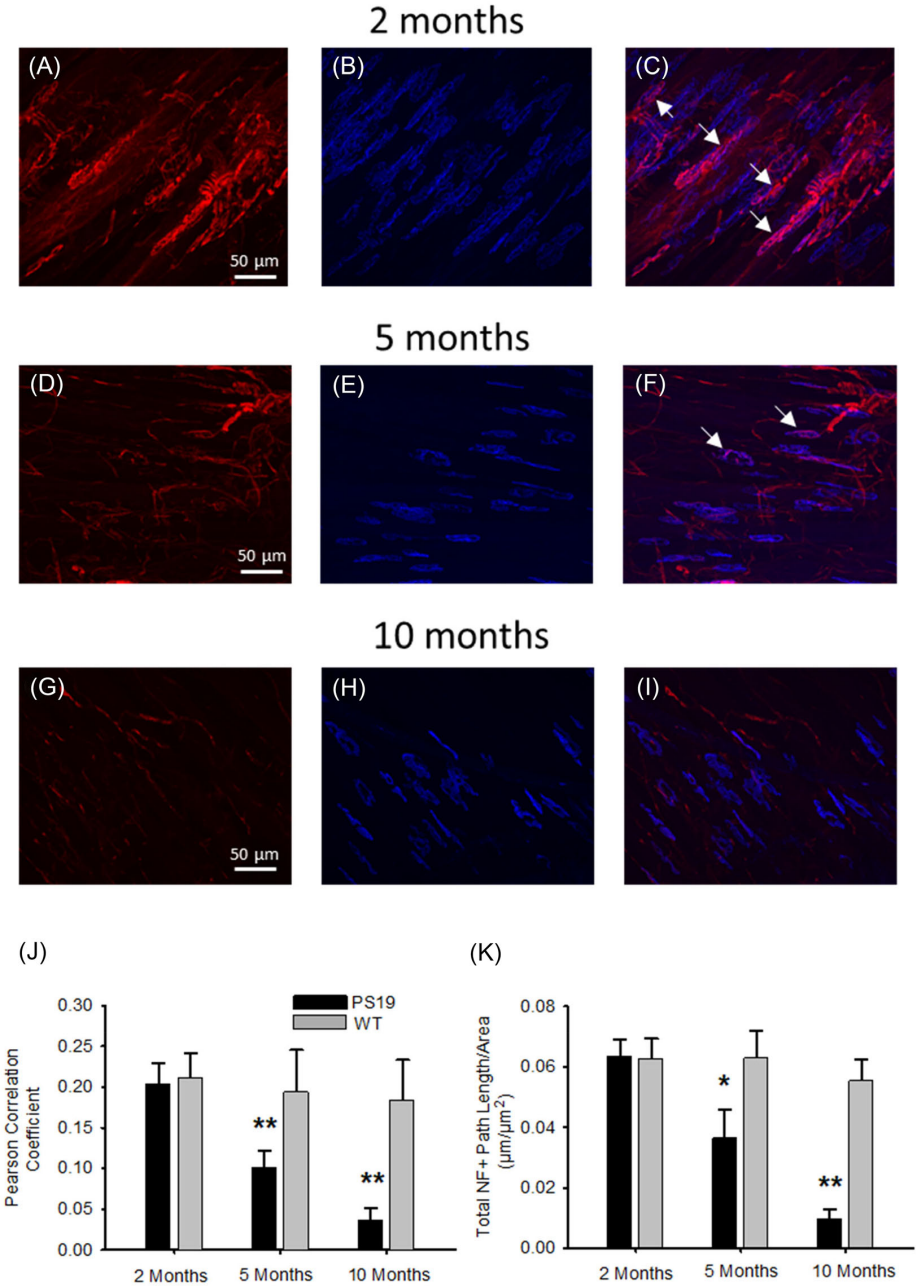
Neuromuscular junction post‐terminal denervation in relation to genotype and age. Organization of NMJs in the lumbricalis muscles of PS19 mice at 2 (A–C), 5 (D–F), and 10 (G–I) months of age. NMJ pre‐terminals were marked with a neurofilament antibody (panels A, D, G) and post‐terminals were highlighted using fluorescent‐labelled α‐bungarotoxin (panels B, E, H). Panels C, F, and I depict the alignment of pre‐ and post‐terminals. Analysis included six mice per genotype and age group, with an equal distribution of 3 males and 3 females; data were combined due to the absence of significant sex‐based differences in both PS19 and WT mice. Each mouse's data represents the average from 20 to 25 NMJs. The evaluation of the spatial correlation between pre‐ and post‐terminals through the Pearson correlation coefficient revealed a marked reduction in myofibre innervation in PS19 mice at 5 and 10 months compared with the 2‐month cohort (**P < 0.001, panel J). Additionally, the total neurofilament‐positive path length, adjusted for the area of study, showed a significant decrease at 5 (*P < 0.05) and 10 months (**P < 0.001) in PS19 mice, a change not observed in WT mice (panel K). Spatial calibration bars are applicable across all images from (A) to (I).

Figure 8 shows the pre‐terminals labelled with a neurofilament antibody (A, D, and G), and the post‐terminals stained with fluorescent‐labelled α‐bungarotoxin (B, E, and H), emphasizing the architecture of the NMJs in lumbricalis muscle. At 2 months, both pre‐ and post‐terminals appeared well‐developed (A and B), with several intact NMJs characterized by overlapping pre‐ and post‐terminals in purple, indicating innervated fibres (C, arrows). However, by 5 months, there was noticeable simplification and disorganization in the pre‐terminals (D), and the post‐terminals were smaller (E), leading to reduced and partially innervated post‐terminals as seen by their overlap (F, arrows). By 10 months, there was dramatic attenuation of pre‐terminals (G) and post‐terminal atrophy (H), culminating in extensive myofibre denervation (I).

To quantify the innervation patterns, we analysed the spatial correlation between pre‐ and post‐terminals using the Pearson correlation coefficient in both PS19 and wild‐type (WT) mice. The PS19 mice exhibited a significant decline in myofibre innervation at 5 and 10 months of age compared with the 2‐month‐old group (J). Additionally, the total path length positive for neurofilament staining also decreased significantly at 5 and 10 months in PS19 mice, but not in WT mice, indicating a specific pattern of denervation in the PS19 model (K).

## Discussion

### The mouse model

To accurately model the neurofibrillary tangles characteristic of tauopathies, including AD, it has been imperative to develop transgenic mice expressing microtubule‐associated protein tau (MAPT) gene alterations. This is despite the absence of specific MAPT mutations in these conditions, except for frontotemporal dementia. For nearly two decades, studies involving MAPT mutations, either alone or in conjunction with amyloid plaques, have shown that overexpression of mutant tau is essential for the development of phospho‐tau pathology and neurofibrillary tangles. Unlike many APP/Aβ models, and in alignment with human clinicopathological findings, tau mouse models demonstrate age‐dependent neurodegeneration, as well as synaptic and cognitive deficits. This suggests a direct neurotoxicity of misfolded and/or aggregated tau. Consequently, the P301S–PS19 model utilized in our study represents a significant step forward in understanding the role of misfolded tau protein in the pathogenesis of tauopathies and the clinical progression and neuropathology of AD.^30^

### Muscle mass reduction with aging in PS19 mice

The reduction in muscle mass observed in the TA, gastrocnemius, EDL, and soleus muscles during aging is prominently influenced by the genotype, along with the interaction of genotype with age, in both male and female PS19 mice, noticeable from 7 to 8 months onwards. This phenomenon highlights the significant impact of genetic factors on muscle atrophy associated with aging. Contrarily, interactions between age and sex did not show marked differences across the examined muscles, leading to an in‐depth morphological examination of myofibres within the EDL and soleus muscles. These muscles are distinguished by their unique fibre type composition and are commonly used in force measurement studies, making them critical for understanding the mechanisms behind muscle atrophy in this context.^31^

### Central nervous system expression of P301S mutation leads to progressive muscle denervation in aging

Our analysis of fibre types in the EDL and soleus muscles demonstrates a significant shift in fibre population distribution in PS19 mice relative to wild types. In male EDL muscles, we observed a gradual decrease in Type IIB fibres, offset by an increase in hybrid fibres. This shift begins at 7–8 months in female EDL muscles, suggesting a significant reorganization of the EDL muscle's cytoarchitecture with aging in PS19 mice.

In PS19 mice's soleus muscle, a notable decrease in Type IIA fibres was observed, together with an increase in Type IIX and hybrid IIA + IIX fibres, starting at 7–8 months of age in males. This contrasted with their younger counterparts and age‐matched WT mice. In females, these changes were delayed, becoming apparent at 9–10 months. Such alterations highlight a genotype and age‐driven myofibre reorganization within the soleus muscle of PS19 mice, indicative of the broader impact of the P301S mutation on muscle composition.

Interestingly, these cytoarchitectural changes in skeletal muscle do not coincide with significant myofibre grouping, possibly due to the disease's accelerated progression, which shortens lifespan to about a third of that seen in standard mouse strains. Such minimal myofibre grouping contrasts with severe spinal cord motoneuron conditions like amyotrophic lateral sclerosis,^29^, ^32^, ^33^ where it is relatively uncommon. Conversely, prominent myofibre grouping is often noted in the skeletal muscles of older adults,^29^ suggesting that myofibre grouping is more evident in conditions with a more gradual progression.

### Neural cell adhesion molecule and neuromuscular junction denervation

In this study, we utilized a morphometric approach that employed NCAM as a histological marker for myofibre denervation. The glycoprotein NCAM (a.k.a. CD56), a member of the Ig superfamily, was originally recognized for its role in neuronal adhesion but is also expressed in muscle cells, regulated by innervation^34^, ^35^ Primarily found on most neural cells, NCAM is crucial in nervous system development, facilitating cell adhesion through its binding with NCAM on other cells. Recent studies have expanded our understanding of NCAM's functions, including cell adhesion, formation of signalling complexes, activation of signalling pathways, cytoskeletal regulation, and growth factor signalling modulation, notably GDNF, BDNF, and PDGF.^36^

In skeletal muscles, NCAM expression is innervation‐dependent, mirroring the muscle's synaptic receptivity.^37^ Abundant in embryonic myotubes during synaptogenesis, it disappears in adulthood but reemerges with denervation. A detailed analysis revealed a significant increase in NCAM protein and mRNA levels upon denervation.^38^

NCAM is present at neuromuscular junctions, within axons, and in non‐innervated satellite cells (SCs). Furthermore, NCAM expression is significantly elevated in the sarcolemma of muscles undergoing denervation, a phenomenon observed in aging,^29^, ^39^, ^40^ ALS, and various experimental models.^38^ This observation is consistent with findings from prior studies.^35^ The specific expression sites of NCAM enable the differentiation of terminal muscle axons and SCs from myofibres that have undergone denervation.

The analysis of the NMJ and NF path length reveals that the changes observed in muscle fibre composition and morphology in PS19 mice, such as the transition from pure myosin heavy chain (MyHC) fibres to hybrid myofibres, and the alterations in cross‐sectional area (CSA) and NCAM immunoreactivity, are likely due to extensive denervation processes impacting NMJ integrity. This denervation becomes significant in PS19 mice at 5 months of age and further intensifies by 10 months. Such changes could explain the observed sarcopenia and premature death in these mice.

### Chronological progression of hyperphosphorylated tau accumulation and its consequences on muscular and cognitive functions

The emergence of motor abnormalities, such as limb clasping and hindlimb retraction during tail suspension, is observed early in PS19 mice's life, preceding neurofibrillary tangle formation in critical brain regions by 6 months. These preliminary motor signs hint at neurogenic alterations in muscle prior to the visible neurofibrillary pathology, aligning with tau aggregate deposition seen in AD and related tauopathies.^21^ Conversely, cognitive deficits, evidenced by delayed spatial task performance in the Morris Water Maze, do not appear until 12 months, indicating subsequent cognitive decline.^30^ ^, S1 41, S2 42^

Hyperphosphorylated tau presence in spinal cord motoneurons^21^ and their projections into the spinal nerves,^S3 43^ suggests that the muscle phenotypes in PS19 mice, also seen in tau knockout^S4 44^ and high P301S mutant tau protein‐expressing mouse models^S5 45^ may involve more than tau pathology alone. The similarity in myelinated axon diameters between PS19 and WT mice^S3 43^ implies that muscle pathology differences stem not from myelin quantity, but likely from compromised axonal transport, due to early tau deposits.

This study's timeline, marked by increasing Type I and II NCAM+ myofibre populations, followed by selective myofibre atrophy and emergence of multi‐MyHC type myofibres, points to early muscle innervation impairment leading to sarcopenia in PS19 mice. This significant neuromuscular dysfunction shortens lifespan dramatically, mirroring age‐related sarcopenia in older mice models, but occurring prematurely in PS19 mice.^24^, ^26^, ^27^ Thus, central nervous system tau deposits expedite neurogenic sarcopenia onset, highlighting a distinct gap between the fast‐paced muscle pathology and the more delayed cognitive decline in the PS19 AD model.

One potential reason for the early and significant impact on the motor system in AD may be the increased susceptibility of the central motor system, notably spinal cord motoneurons, to accumulations of misfolded tau protein. Changes in muscle mass, gait, and overall mobility could be more apparent than reductions in learning and memory capabilities, which are frequently attributed to the normal aging process.

## Conflict of interest

The authors declare no conflicts of interest.

## Supporting information


**Table S1** Percentage of Fibre Grouping in Soleus Muscle
**Figure S1:** Correlation of Actual and Predicted Muscle Weights in Mice by Muscle Type. (A) TA Muscle, (B) GA Muscle, (C) EDL Muscle, and (D) Soleus Muscle. Each plot displays individual data points representing the actual muscle weight plotted against the predicted values, with the line of best fit indicating the correlation. The degree of correlation is quantified by the R‐squared value, with TA and EDL muscles showing moderate correlations (R‐squared = 0.63), GA muscle showing a stronger correlation (R‐squared = 0.71), and Soleus muscle having a slightly lower correlation (R‐squared = 0.59). All plots demonstrate statistically significant relationships (*P* < 0.0001), emphasizing the predictive model's effectiveness. In each plot, the regression line and the shaded area indicating the confidence interval suggest that the predictive model has a reliable degree of accuracy in estimating muscle weight based on the given variables, with the Root Mean Square Error (RMSE) providing a measure of the average deviation of the predictions from the actual values. The RMSE values indicate the model's precision in prediction, with lower values suggesting closer agreement between actual and predicted weights. The shaded areas represent 95% confidence intervals for the regression lines, providing a visual representation of the model's accuracy. The consistency in statistical significance across all muscle types suggests the robustness of the predictive approach used.

## Data Availability

All data supporting the findings of this study will be made available upon request directed to the corresponding author.
