# Supplementary material for: Accelerated sarcopenia precedes learning and memory impairments in the P301S mouse model of tauopathies and Alzheimer's disease
Source: J Cachexia Sarcopenia Muscle. 2024 Apr 22;15(4):1358–75. doi: 10.1002/jcsm.13482 (PMC11294019; doi:10.1002/jcsm.13482)
Supplement: Supplementary file 1 — Table S1 Percentage of Fibre Grouping in Soleus Muscle Figure S1: Correlation of Actual and Predicted Muscle Weights in Mice by Muscle Type. (A) TA Muscle, (B) GA Muscle, (C) EDL Muscle, and (D) Soleus Muscle. Each plot displays individual data points representing the actual muscle weight plotted against the predicted values, with the line of best fit indicating the correlation. The degree of correlation is quantified by the R‐squared value, with TA and EDL muscles showing moderate correlations (R‐squared = 0.63), GA muscle showing a stronger correlation (R‐squared = 0.71), and Soleus muscle having a slightly lower correlation (R‐squared = 0.59). All plots demonstrate statistically significant relationships (P < 0.0001), emphasizing the predictive model's effectiveness. In each plot, the regression line and the shaded area indicating the confidence interval suggest that the predictive model has a reliable degree of accuracy in estimating muscle weight based on the given variables, with the Root Mean Square Error (RMSE) providing a measure of the average deviation of the predictions from the actual values. The RMSE values indicate the model's precision in prediction, with lower values suggesting closer agreement between actual and predicted weights. The shaded areas represent 95% confidence intervals for the regression lines, providing a visual representation of the model's accuracy. The consistency in statistical significance across all muscle types suggests the robustness of the predictive approach used. [file JCSM-15-1358-s001.docx]

**SUPPLEMENTARY INFORMATION**

**Accelerated Sarcopenia Precedes Learning and Memory Impairments in the P301S Mouse Model of Tauopathies and Alzheimer’s Disease**

Savannah Longo, María Laura Messi, Zhong-Min Wang, William Meeker,

and Osvaldo Delbono

Department of Internal Medicine, Sections on Gerontology and Geriatric Medicine.

Wake Forest University School of Medicine, Winston-Salem, NC 27157

**References Supplementary to the Main Text**

S1 41. Ahmad F, Mein H, Jing Y, Zhang H, Liu P. Behavioural Functions and Cerebral Blood Flow in a P301S Tauopathy Mouse Model: A Time-Course Study. International Journal of Molecular Sciences. 2021;22:9727.

S2 42. Yoshiyama Y, Kojima A, Ishikawa C, Arai K. Anti-Inflammatory Action of Donepezil Ameliorates Tau Pathology, Synaptic Loss, and Neurodegeneration in a Tauopathy Mouse Model. Journal of Alzheimer's Disease. 2010;22:295-306.

S3 43. Merchán-Rubira J, Sebastián-Serrano Á, Díaz-Hernández M, Avila J, Hernández F. Peripheral nervous system effects in the PS19 tau transgenic mouse model of tauopathy. Neuroscience Letters. 2019;698:204-8.

S4 44. Lopes S, Lopes A, Pinto V, Guimarães MR, Sardinha VM, Duarte-Silva S, et al. Absence of Tau triggers age-dependent sciatic nerve morphofunctional deficits and motor impairment. Aging Cell. 2016;15:208-16.

S5 45. Yin Z, Valkenburg F, Hornix BE, Mantingh-Otter I, Zhou X, Mari M, et al. Progressive motor deficit is mediated by the denervation of neuromuscular junctions and axonal degeneration in transgenic mice expressing mutant (P301S) tau protein. Journal of Alzheimer's Disease. 2017;60:S41-S57.

**Supplementary Table 1**

| **Genotype** | **Sex** | **Age**  **(months)** | **Type-I** | **Type-II** |
| --- | --- | --- | --- | --- |
| **Negative** | Male | 1-2 | 0.000±0.000 | 0.000±0.000 |
|  | Female | 1-2 | 0.000±0.000 | 0.000±0.000 |
|  | Male | 3-4 | 0.000±0.000 | 2.520±1.787 |
|  | Female | 3-4 | 0.000±0.000 | 0.000±0.000 |
|  | Male | 5-6 | 0.000±0.000 | 2.192±0.012 |
|  | Female | 5-6 | 1.333±1.333 | 0.000±0.000 |
|  | Male | 7-8 | 0.000±0.000 | 2.431±1.427 |
|  | Female | 7-8 | 0.000±0.000 | 0.000±0.000 |
|  | Male | 9-10 | 0.000±0.000 | 2.523±1.292 |
|  | Female | 9-10 | 1.513±1.543 | 1.641±1.641 |
|  | Male | 11-12 | 0.000±0.000 | 5.135±3.795 |
|  | Female | 11-12 | 1.899±1.124 | 1.356±0.855 |
|  |  |  |  |  |
| **Positive** | Male | 1-2 | 0.000±0.000 (ns) | 0.000±0.000 (ns) |
|  | Female | 1-2 | 0.000±0.000 (ns) | 0.000±0.000 (ns) |
|  | Male | 3-4 | 0.000±0.000 (ns) | 4.462±3.246 (ns) |
|  | Female | 3-4 | 0.000±0.000 (ns) | 0.000±0.000 (ns) |
|  | Male | 5-6 | 0.000±0.000 (ns) | 2.208±1.278 (ns) |
|  | Female | 5-6 | 2.551±1.489 (ns) | 0.000±0.000 (ns) |
|  | Male | 7-8 | 0.000±0.000 (ns) | 1.975±1.975 (ns) |
|  | Female | 7-8 | 1.506±1.506 (ns) | 1.335±1.335 (ns) |
|  | Male | 9-10 | 0.000±0.000 (ns) | 7.875±3.859 (ns) |
|  | Female | 9-10 | 2.438±1.710 (ns) | 0.000±0.000 (ns) |
|  | Male | 11-12 | 0.000±0.000 (ns) | 0.760±0.760 (ns) |
|  | Female | 11-12 | 0.000±0.000 (ns) | 0.000±0.000 (ns) |

**Percentage of Fiber Grouping in Soleus Muscle**

Values are presented as mean ± SEM. NS denotes no statistically significant

difference between genotypes and age groups.


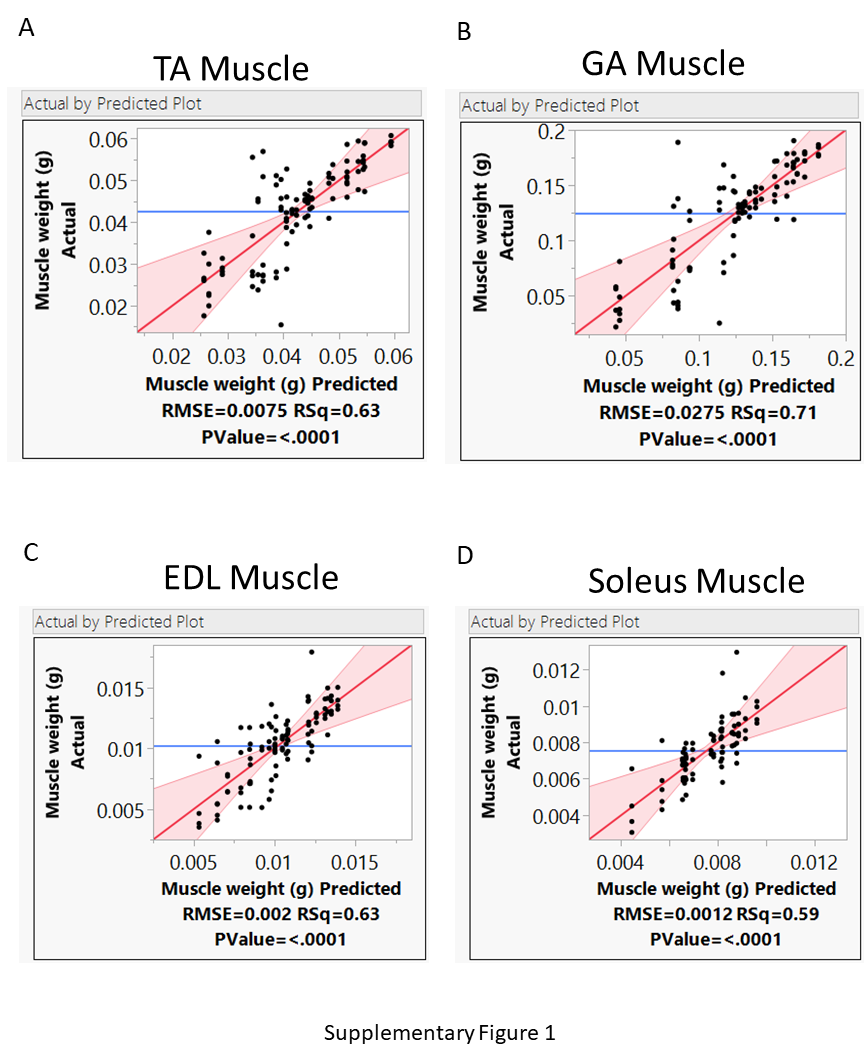


**Supplementary Figure Legend**

**Supplementary Figure 1: Correlation of Actual and Predicted Muscle Weights in Mice by Muscle Type. (A) TA Muscle, (B) GA Muscle, (C) EDL Muscle, and (D) Soleus Muscle.** Each plot displays individual data points representing the actual muscle weight plotted against the predicted values, with the line of best fit indicating the correlation. The degree of correlation is quantified by the R-squared value, with TA and EDL muscles showing moderate correlations (R-squared = 0.63), GA muscle showing a stronger correlation (R-squared = 0.71), and Soleus muscle having a slightly lower correlation (R-squared = 0.59). All plots demonstrate statistically significant relationships (P < 0.0001), emphasizing the predictive model's effectiveness. In each plot, the regression line and the shaded area indicating the confidence interval suggest that the predictive model has a reliable degree of accuracy in estimating muscle weight based on the given variables, with the Root Mean Square Error (RMSE) providing a measure of the average deviation of the predictions from the actual values. The RMSE values indicate the model's precision in prediction, with lower values suggesting closer agreement between actual and predicted weights. The shaded areas represent 95% confidence intervals for the regression lines, providing a visual representation of the model's accuracy. The consistency in statistical significance across all muscle types suggests the robustness of the predictive approach used.

**Material and Methods**

**Mouse model**

P301S mouse colony founders were purchased to Jackson Labs (Bar Harbor, ME). Mouse genotyping followed Jackson Labs’ protocols. Mice were kept in a pathogen-free area of the Wake Forest University School of Medicine (WFUSOM) Animal Research Program at 21°C and a 12:12 h dark/light cycle until the day of the experiment.  The mice had *ad libitum* access to standard chow and continuous access to drinking water throughout the study.

**Muscle weight**

Muscles were meticulously dissected under a stereoscope using fine dissecting tools immediately following mouse euthanasia. The muscles were blotted, and the tendons were carefully trimmed close to the longest muscle fibers, ensuring not to sever them. Any adherent blood and fat were thoroughly removed. The cleaned muscles were then weighed on a precision scale and subsequently processed for immunohistochemistry.

**Skeletal Muscle Immnohistochemistry**

***Skeletal Muscle Histochemical and Immunofluorescence Analyses***

Skeletal muscle histochemical and immunofluorescence analyses followed reported procedures,[1] with some modifications. Briefly, muscle samples were carefully dissected, and visible blood and connective tissue removed. Each muscle specimen was then oriented perpendicularly on a 35 mm dish coated with Sakura Tissue Tek compound (Ted Pella, Redding, CA) optimum cutting temperature (O.C.T.) thickened with baby powder. Utilizing fine tweezers under a stereomicroscope, one end of the muscle was carefully dipped into the medium, allowing for half of the muscle to remain exposed, and then promptly subjected it to flash freezing in liquid nitrogen. This freezing technique prevents the formation of ice crystals that can disrupt cellular structures and negatively impact subsequent staining. After freezing, the specimens were transferred to a -80°C freezer for storage, keeping the muscle in an optimally preserved state until sectioning. Sectioning was performed at -20°C using a Leica CM3050S cryostat (Teaneck, NJ), carefully slicing the muscle into 10-µm thick sections. Post-sectioning, the samples were kept at -20°C to retain tissue quality up to the point of staining and analysis.

***ATPase Staining and NCAM Immunofluorescence***

Initially, cryosections underwent preincubation in a basic solution (pH 9.4) to selectively inhibit ATPase activity in type I (slow-twitch) fibers, effectively differentiating them from type II (fast-twitch) fibers upon subsequent staining.

Following preincubation, sections were washed in phospho-buffered saline (PBS) and then subjected to ATPase staining. To preserve the enzymatic activity and maintain tissue morphology, the sections were fixed in 2% paraformaldehyde (PFA). After fixation, sections were permeabilized with PBS containing 0.1% Triton X-100 (PBST) and non-specific binding sites were blocked using 5% goat serum in PBS, ensuring specificity of antibody binding. The sections were then incubated with a primary antibody mixture containing laminin antibody (MAB 1914P Millipore, Sigma; dilution 1:200), 1% goat serum, and NCAM (Sigma-Millipore, Cat #AB5032; 1:200) in PBS. This incubation was conducted in a histology tray at 4°C overnight to facilitate optimal antibody-antigen interaction.

The following day, after thorough washing in PBST to remove unbound primary antibodies, the sections were exposed to fluorescently labeled secondary antibodies. These included Alexa Fluor (AF) goat anti-rat IgG 680 Cy5 (A21096; 1:500) for laminin detection and AF goat anti-rabbit IgG 488 (Cat 11008; 1:1000) for NCAM visualization. The secondary antibodies, also in a solution with 1% goat serum, were applied in a histology tray and incubated overnight at 4°C, promoting maximal fluorescent conjugation without compromising tissue integrity.

On the third day, the slides were given a final wash in PBS to remove excess secondary antibodies and mounted using a fluorescence-enhancing mounting medium (S3023 Dako, Carpinteria, CA). The prepared slides were stored at 4°C to preserve fluorescence and prevent photobleaching until they were scanned using a fluorescence microscope.

***Specific MyHC Antibody-Based Immunofluorescence to Identify Myofiber Subtypes***

Myofiber subtype characterization was based on identifying specific myosin heavy chain (MyHC) isoforms. Muscle sections were rinsed in PBST and blocked with 10% goat serum (Cat. # 005-000-121, Jackson Immunoresearch) in PBS for 1 hour at room temperature (RT). Immunofluorescence analysis of MHC expression is performed with a cocktail of primary MHC antibodies from the Developmental Studies Hybridoma Bank (DSHB), University of Iowa: MyHC-I (concentrate BA-F8 Ab; dilution 1:50), MHC-IIa (concentrate SC-71, dilution 1:500), MHC-IIb (concentrate BF-F3; dilution 1:100), and Laminin (MAB 1914P Millipore, Sigma; dilution 1:100) and 10% goat serum in PBS for 2 hours at RT. After washing the sections with PBST, they were incubated with the secondary antibodies AF goat anti-mouse; IgG2b 350 blue; A21140; dilution (dil) 1:500; for MHC-I, AF goat anti-mouse; IgG 488 green; A21121; dil 1:500; for MHC-IIa), AF goat anti-mouse; IgM 555 red; A21426; dil 1:500; for MHC-IIb, AF goat anti-rat; IgG 680 Cy5; A21096; dil 1:500 for Laminin, plus 10% goat serum in PBS for 1.5 h at RT. All secondary antibodies were purchased to Invitrogen, Thermo Fisher (Carlsbad, CA). Samples were washed in PBS and Tissue sections were mounted using fluorescence mounting medium (Dako) and visualized with an inverted, motorized, fluorescent microscope (Olympus IX81, Tokyo, Japan) with an Orca-R2 Hamamatsu CCD camera (Hamamatsu, Japan). The camera driver and image acquisition were controlled with a MetaMorph Imaging System (Olympus).[2] This approach was previously validated by staining serial sections with an antibody specific for type-IIx fibers (6H1, University of Iowa, Developmental Study Hybridoma Bank). Fibers immunoreactive to 6H1 antibody correspond to unstained fibers exposed to the antibody cocktail described above.[1, 2]

**Image Analysis**

Digital histological images were acquired at 20X magnification for detailed analysis. We performed image quantification by using a custom-made script, which runs within the NIH ImageJ/Fiji software environment, encompassed four key steps: (1) delineating muscle fibers using laminin-based segmentation, (2) implementing an editing sequence to refine the segmentation, (3) conducting fiber type thresholding and analysis, and (4) applying an editing sequence for accurate fiber typing. This semiautomatic script enabled corrections for potential errors in both image segmentation and fiber type classification. This approach combined speed and precision in muscle morphometry analysis, incorporating operator intervention to ensure the reliability and reproducibility of the results. [3] In our original communication, we validated the computer-based analysis through a manual examination.[3]

**NMJ Analysis in Lumbricalis Muscle Whole Mount Preparation**

For NMJ analysis, lumbricalis muscles were carefully dissected and isolated from adjacent tissues. Muscles were then fixed onto Sylgard 184 silicone-coated dishes (Dow Corning, Midland, MI) using pins at the tendons, fixed overnight in 2% PFA at 4°C, and subsequently washed three times in PBST. Blocking was performed with a solution of 1% Triton X-100 and 10% goat serum in PBS at 4°C overnight. The following day, staining was carried out for nerve neurofilament and synaptic vesicle protein 2 using anti-neurofilament SMI 311 (Biolegend, San Diego, CA) and SV2 (Developmental Studies Hybridoma Bank, Iowa City, IA) antibodies, respectively, along with α-bungarotoxin (BGT) CF680R (Biotium, Fremont, CA) in 1% Triton X-100 and 4% goat serum in PBS at 4°C overnight. After three washes in PBST, an AF568 goat anti-mouse IgG (Thermo Fisher) was applied as the secondary antibody for SMI-311 and SV2, together with 1% goat serum at room temperature for 4 hours. Following three final washes in PBS, tendons were removed, and the muscle was mounted on a glass slide with Dako mounting medium for visualization at 40X magnification under an Olympus FV1200 spectral laser scanning confocal microscope.

**Statistical Analysis**

Our investigation entailed a complex analysis of muscle weights, NCAM expression levels, myofiber type quantities, cross-sectional areas, and the percentage distribution of various fiber types in PS19 transgenic and wild-type (WT) mice over time. Factorial ANOVA provided insights into the primary effects of each individual factor and their interactive influences. Ensuring the integrity of our analysis, we verified that our dataset met the assumptions necessary for the application of ANOVA, including normality in the distribution of data and homogeneity of variances across the comparative groups. Upon identifying statistically significant outcomes, we proceeded with post-hoc analyses (Tukey’s Honestly Significant Difference or Bonferroni Correction) to elucidate the specific differences between the groups, offering a detailed examination of our findings.

We utilized a least squares model to evaluate statistical differences in outcomes across time, genotype, sex, and muscle types. This approach not only enabled the prediction of outcomes based on our independent variables but also allowed for the simultaneous consideration of multiple covariates, thereby enhancing our understanding of their unique effects.

Our analysis employed the least squares method to derive predicted values, establishing a regression line through observed data points. This approach aims to minimize the sum of squared vertical discrepancies (residuals) between actual observations and model predictions. By applying a linear equation, we delineate the relationship between independent (predictor) and dependent (response) variables. The method fine-tunes model parameters, such as slope and intercept, ensuring the minimized sum of squared differences between observed and predicted values. The optimized equation thus represents the data's best fit line, facilitating the calculation of predicted values for specific independent variables. This meticulous process diminishes the overall deviation between real data points and the model's predictions, rendering the model an effective predictive tool.

The analysis was conducted utilizing Microsoft Excel (Redmond, WA) along with the advanced analytical capabilities of JMP-SAS software (Cary, NC).

**References**

1. Rodrigues ACZ, Messi ML, Wang Z-M, Abba MC, Pereyra A, Birbrair A, et al. The Sympathetic Nervous System Regulates Skeletal Muscle Motor Innervation and Acetylcholine Receptor Stability Acta Physiologica. 2018;225:e13195.

2. Bloemberg D, Quadrilatero J. Rapid determination of myosin heavy chain expression in rat, mouse, and human skeletal muscle using multicolor immunofluorescence analysis. PLoS One. 2012;7:e35273.

3. Bonilla HJ, Messi ML, Sadieva KA, Hamilton CA, Buchman AS, Delbono O. Semiautomatic morphometric analysis of skeletal muscle obtained by needle biopsy in older adults. GeroScience. 2020;6:1628-60.
